# Combined Behavioral and Mismatch Negativity Evidence for the Effects of Long-Lasting High-Definition tDCS in Disorders of Consciousness: A Pilot Study

**DOI:** 10.3389/fnins.2020.00381

**Published:** 2020-04-28

**Authors:** Xiaoyu Wang, Yongkun Guo, Yunge Zhang, Jinju Li, Zhongqi Gao, Yingxin Li, Tianlin Zhou, Hui Zhang, Jianghong He, Fengyu Cong

**Affiliations:** ^1^School of Biomedical Engineering, Faculty of Electronic Information and Electrical Engineering, Dalian University of Technology, Dalian, China; ^2^Department of Neurosurgery, Zhengzhou Central Hospital, Zhengzhou, China; ^3^Department of Neurosurgery, The Fifth Affiliated Hospital of Zhengzhou University, Zhengzhou, China; ^4^Department of Neurosurgery, People’s Liberation Army General Hospital, Beijing, China; ^5^Faculty of Information Technology, University of Jyväskylä, Jyväskylä, Finland

**Keywords:** high-definition transcranial direct current stimulation, disorder of consciousness, coma recovery scale-revised, mismatch negativity, event-related potentials

## Abstract

**Objective:**

To evaluate the effects of long-term High-definition transcranial direct current stimulation (HD-tDCS) over precuneus on the level of consciousness (LOC) and the relationship between Mismatch negativity (MMN) and the LOC over the therapy period in patients with Disorders of consciousness (DOCs).

**Methods:**

We employed a with-in group repeated measures design with an anode HD-tDCS protocol (2 mA, 20 min, the precuneus) on 11 (2 vegetative state and nine minimally conscious state) patients with DOCs. MMN and Coma Recovery Scale-Revised (CRS-R) scores were measured at four time points: before the treatment of HD-tDCS (T0), after a single session of HD-tDCS (T1), after the treatment of 7 days (T2) and 14 days (T3). A frequency-deviant oddball paradigm with two deviation magnitudes (standard stimulus: 1000 Hz, small deviant stimuli: 1050 Hz, large deviant stimuli: 1200 Hz) was adopted to elicit MMN.

**Results:**

Significant improvements of CRS-R score were found after 7-day (T2) and 14-day (T3) treatment compared with baseline (T0). Regarding the MMN, significant improvements of MMN amplitudes were observed after a single session of stimulation (T1), 7-day (T2) and 14-day treatment (T3) compared with baseline (T0). Additionally, there were significant negative correlations between CRS-R scores and MMN amplitudes elicited by both large and small deviant stimuli.

**Conclusion:**

Long-term HD-tDCS over precuneus might improve signs of consciousness in patients with DOCs as measured by CRS-R total scores, and MMN could be an assistant assessment in the course of tDCS treatment.

## Introduction

Disorder of consciousness (DOCs) are clinical states where consciousness and reactivity to external stimuli have impaired by severe brain injury (such as traumatic brain injury, stroke, Hemorrhage, and so forth). Based on the behavioral observations, patients who suffered from DOCs could be categorized as vegetative state/unresponsive wakefulness syndrome (VS/UWS) and minimally conscious state (MCS) group (Giacino et al., 2004). VS is a clinical condition of complete unawareness of the self and environment, and MCS is distinguished from VS by the partial preservation of conscious awareness. MCS can be subcategorized into a lower (MCS-, the presence of visual pursuit, localization of noxious stimulation and/or appropriate smiling/crying) and a higher (MCS +, the presence of command following) level based on the complexity of patients’ behaviors (Bruno et al., 2011). The above clinical assessments mostly follow the Coma Recovery Scale-Revised (CRS-R) scale (Giacino et al., 2004), a standardized behavioral assessment for determining the level of consciousness (LOC) in patients with DOCs. Regarding treatment, peripheral treatment (e.g., physical therapy, speech therapy), pharmacological (i.e., amantadine, zolpidem) and non-pharmacological interventions (i.e., deep brain stimulation, spinal cord Stimulation) have been investigated in the last decade, however, there remain few effective therapies for patients with DOCs (Schiff et al., 2007; Della Pepa et al., 2013; Angelakis et al., 2014; Cossu, 2014; Tucker and Sandhu, 2016). Current evidence suggests that non-invasive brain stimulation (NIBS) including transcranial direct current stimulation (tDCS), Transcranial magnetic stimulation (TMS), and low-level laser therapy (LLLT), seems to be promising treatments (Thibaut et al., 2014; Xia et al., 2017; Poiani et al., 2018; Huang et al., 2019). For instance, Xia et al. (2017) reported that 10 Hz multisession repetitive TMS applied to the left dorsolateral prefrontal cortex (DLPFC) has a potential benefit for the rehabilitation of patients with severe DOC, with an increase in CRS-R total scores in 5 out 5 MCS patients and 4 out of 11 VS/UWS patients after 30-day treatment. In addition, Poiani et al. (2018) reported that the design of a randomized double-blinded trial of photobiomodulation using LLLT could be an effective treatment for patients with traumatic brain injury (TBI). Notably, tDCS has received considerable attention in the field of neuroscience because of its low cost, portability, safety, tolerance (Bikson et al., 2016) and combination with robotic-based rehabilitation (Calabro et al., 2016).

tDCS uses a weak constant current which flows through the brain from the anode to the cathode to alter cortical excitability, and performance improvements have been observed following anodal stimulation of brain regions associated with visual, motor (Calabro et al., 2016), and auditory processing functions in healthy subjects (Impey et al., 2017), whereas cathodal tDCS can reduce cortical excitability (Lefaucheur et al., 2017). Additionally, several studies have shown that tDCS can transiently improve the LOC of patients with DOCs as measured by changes in CRS-R total scores (Angelakis et al., 2014; Thibaut et al., 2014, 2017). However, the main drawback of conventional tDCS is that it produces diffuse brain current flow, which makes it difficult to conclude a precise cortical region producing clinical effects. High-Definition tDCS (HD-tDCS) using the 4 × 1 smaller compact scalp electrodes improves the spatial resolution and focus the electric field, which is believed to overcome the above drawback and enhance the clinical outcomes (Castillo-Saavedra et al., 2016). There are few studies have examined the impact of HD-tDCS on DOC to date (Guo et al., 2019). In the present study, we used HD-tDCS to increase focality of the stimulation.

A critical issue of tDCS in patients with DOC is the stimulated brain region. Several clinical studies have been conducted over different stimulation areas in patients with DOCs to investigate the efficacy. The DLPFC was the most used target, which was thought to be involved in many high cognitive processes such as attention, planning, decision making (Dockery et al., 2009; Kang et al., 2012) and others. For instance, Thibaut et al. (2014) found that a single-session anodal tDCS applied to the left DLPFC improved CRS-R total scores in MCS patients without side effects, and Angelakis et al. (2014) reported that similar treatment improvement of CRS-R total scores was found under a multi-session tDCS over the left DLPFC. However, DLPFC is likely to be damaged in TBI, which is currently the most common neurologic cause leading to loss of consciousness (Bekinschtein et al., 2015). Similarly, precuneus is also a critical region for consciousness recovery (Laureys and Schiff, 2012), where notably increased blood flow is associated with the emergence from anesthesia (Xie et al., 2011). Besides, the precuneus and medial prefrontal cortex are linked to the default mode network (DMN), where connectivity decreased in patients with DOCs (Norton et al., 2012; Koenig et al., 2014). Alternatively, Huang et al. (2017) stimulated the posterior parietal cortex/precuneus and found positive improvements in the CRS-R scores, and this observation was latter matched by our recent pilot study (Guo et al., 2019). However, the clinical effects of HD-tDCS over the precuneus in patients with DOCs have not been fully investigated, especially, the electrophysiological evidence. Based on the above literature, we chose the precuneus as the stimulated area in this study.

Determining the LOCs in patients with DOCs plays a vitally important role in evaluating the treatment effect, and it predicts the awakening from coma. At present, in clinical practice, such diagnosis is mainly based on a set of clinical observations. In terms of clinical variables, the CRS-R scale with the total score ranges between 0 (coma) and 23 (emergence from MCS), includes auditory, visual, motor, oromotor, communication, and arousal functions. Nonetheless, this approach is pretty subjective and has a low resolution in assessing consciousness, which leads to high diagnostic errors (Andrews et al., 1996). Currently, functional magnetic resonance imaging (fMRI) and electroencephalography (EEG) techniques have enabled us to investigate the brain structure and function in patients with DOCs (Harrison and Connolly, 2013). However, the drawbacks of fMRI-based DOCs studies are expensive and inconvenient which can’t be performed at the patients’ bedsides. EEG technique circumvents nearly all of the portability, cost, and data acquisition issues of fMRI (Bai et al., 2017). To facilitate clinical diagnosis, Event-Related Potentials (ERPs), mainly including Mismatch Negativity (MMN) and P300, have been widely investigated in the last two decades. Compared with P300 component requiring higher cognitive capacities (attentive resources), MMN is a relatively automatic (pre-attentive) response to an occasional mismatched deviant stimulus that differs from repeated standard stimuli and can be used to assesses auditory discriminations, representing an early sensory-memory trace formation (Näätänen et al., 2007; Impey et al., 2017). Besides, MMN can be measured under both attended and unattended conditions (Näätänen et al., 2007), implying that it could also be successfully elicited in patients with DOCs. Impey et al. (2017) reported that anodal tDCS over the temporal cortex increased MMN-indexed auditory detection of pitch deviance in healthy adults, which suggests that treatment and assessment combining MMN and tDCS techniques should be further explored in patients with DOCs.

At present, frequency and duration deviants are mostly employed in patients with DOCs (Tzovara et al., 2012; King et al., 2013; Risetti et al., 2013), particularly, Frodl-Bauch et al. (1997) reported that frequency-deviant paradigms elicited stable MMN components. Due to the impairment of consciousness, a large frequency deviance, such as 200, 500, and 1000 Hz (Naccache et al., 2005; Wijnen et al., 2007; Tzovara et al., 2012), is applied in patients in DOCs, whereas a small one, such as 16, 32, and 50 Hz (Sams et al., 1985; Impey et al., 2017), is used among healthy individuals, which exceeds the healthy subject’s discrimination threshold. The stimulus deviance is a critical scientific issue, and it should be at the same time perceptually well discriminable but not so large as to elicit considerable feature-specific neuronal activity which may cause MMN partially overlapped with N1 and/or N2b components (Tervaniemi et al., 1999). We hypothesized that a small deviant stimulus is suitable for assessing the patients with a higher LOC without overlapping effects, and a large deviant stimulus is proper for diagnosing the patients with a lower LOC to exceed the discrimination threshold. Still, no study evaluates whether the above small frequency deviance can be detected as the auditory discrimination altered under a specific treatment in patients with DOCs.

In this study, we employed a with-in group repeated measures design. All patients received HD-tDCS modulation over precuneus for two sessions per day over 14 consecutive days. The behavioral (CRS-R) and electrophysiological (MMN) assessments were measured at four time points: before the treatment of HD-tDCS (T0), after a single session of HD-tDCS (T1), after the treatment of 7 days (T2) and 14 days (T3). We aim to investigate (1) the effects of long-term anodal HD-tDCS in patients with DOCs as assessed by CRS-R scores and MMN features respectively, namely the treatment improvement on CRS-R scores or MMN features in patients with DOCs; (2) the correlation between the clinical assessments performed by CRS-R scale and MMN features over the therapy period, that is to say, the potential of using MMN features as a clinically electrophysiological assessment under specific treatment.

## Materials and Methods

### Patients and Controls

Fourteen patients with severe brain injury were consecutively recruited from the Department of Neurosurgery, Zhengzhou Central Hospital Affiliated to Zhengzhou University, between January 2018 and August 2018. Experienced neurologists rated each subject using the CRS-R scale (Giacino et al., 2004). Inclusion criteria were being VS/UWS or MCS patients according to the CRS-R scores. As for patient management, the patients diagnosed as DOCs (the coma interval since the event to 28^th^ day would be used to confirm diagnosis) received about 4-week routine medications and rehabilitation courses after admission to the hospital. And if no CRS-R score change was found during the interval, they would receive tDCS stimulation for further treatment (Patient #1–2, #4–8, #10–12, and #14, detail in Table 1). Three enrolled patients didn’t satisfy the above treatment procedures. Patient #3 received tDCS stimulations on the 9^th^ day after admission to hospital since she had completed the 4-week routine medications period before transformed from the other hospital; patient #9 only got a 12-day routine medications and rehabilitation courses; patient #13 received tDCS stimulations on the 8^th^ day after admission to hospital since the extremely low level of consciousness (CRS-R score = 2). We excluded patients who had precuneus lesions, and showed an obvious increase or decrease in consciousness 1 week prior to the HD-tDCS treatment. Participants who had pacemakers, aneurysm clips, other devices implanted or other treatments and drugs which modifying cortical-excitability were also eliminated. Additionally, regarding the MMN-based index, we excluded patient who was absent from the N1 component in the individual raw ERP averaged waveform, which is the pre-condition of measuring MMN (see section “Criterions of identifying and quantifying MMN properties”). Consequently, 11 patients (2VS and 9MCS-, six females and five males, patients #12, #13, and #14 were excluded due to the absence of the N1 component in the grand averaged waveform) aged between 32 and 70 years old (mean 54.2 ± 13.1) were enrolled to complete the entire experiment, detail in Table 1.

**TABLE 1:** The detail information about patients.

**Patient**	**Gender (M, F)**	**Age (Years)**	**Etiology**	**Interval since event**	**Interval of no CRS-R score change since admission to hospital**	**Total CRS-R score**	**Diagnosis**
1	M	48	TBI	58 days	28 days	6	MCS-
2	M	52	Hemorrhage	90 days	30 days	10	MCS-
3	F	38	Hemorrhage	320 days	9 days	8	MCS-
4	F	56	Hemorrhage	56 days	28 days	9	MCS-
5	F	62	Hemorrhage	115 days	28 days	6	MCS-
6	M	32	TBI	64 days	31 days	4	VS
7	M	41	Hemorrhage	48 days	29 days	4	VS
8	M	70	Hemorrhage	76 days	28 days	13	MCS-
9	F	69	Stroke	40 days	12 days	12	MCS-
10	M	67	Hemorrhage	82 days	29 days	10	MCS-
11	F	61	Hemorrhage	75 days	28 days	16	MCS-
12	F	66	Hemorrhage	73 days	30 days	7	MCS-
13	M	52	Hemorrhage	45 days	8 days	2	VS
14	M	60	Cerebral Anoxia	115 days	30 days	4	VS

*According to the previous study, the MCS patients were subcategorized into MCS- (i.e., patients only showing non-reflex behavior such as visual pursuit, localization of noxious stimulation and/or contingent behavior) and MCS + (i.e., patients showing command following) in our study. TBI means Traumatic Brain injury.*

Based on previous literature (Tzovara et al., 2012), to evaluate whether the MMN component could be successfully elicited by our paradigm and to set the criterions of identifying and quantifying MMN, we additionally collected the EEG data of four age-matched healthy volunteers as a control group using the same paradigm at T0. None had a history of neurological or psychiatric illnesses, and all reported normal hearing. No quantitative comparison was performed between auditory discrimination of patients and controls. Written informed consents were acquired from all the patients’ families and caregivers. The present study was conducted according to the Declaration of Helsinki, and approved by the ethics committee of the Zhengzhou Central Hospital Affiliated to Zhengzhou University.

### HD-tDCS Protocol

We employed an HD-tDCS device (Model 4 × 1-C2: Soterix Medical Inc., New York, NY) using 4 × 1-Ring high-definition electrodes with an anode center electrode overlying the targeted brain area surrounded by four cathodal electrodes to deliver direct current to the scalp via Ag/AgCl sintered ring electrodes. Electrodes were held in place by specially designed plastic casings embedded in a 32-channel EEG recording cap which was also used to house EEG recording electrodes during data acquisition. The anode was placed at Pz according to the 10/20 International System, and four cathodal return electrodes were placed approximately 3.5 cm radially from Pz; corresponding roughly to locations Cz, P3, P4, and POz. In this study, all patients received HD-tDCS modulation with anode centered on the precuneus for two sessions each day in the afternoon over 14 consecutive days. During the HD-tDCS session, the direct current was gradually increased to 2 mA, which was constantly delivered for 20 min. The HD-tDCS treatment was administered to the patients in their hospital beds, and any side effects of HD-tDCS were monitored and recorded.

### Outcomes

The primary research question was whether long-lasting anodal HD-tDCS, as compared to baseline (before the HD-tDCS intervention), would improve consciousness. The outcomes of patients with DOCs were single-blind determined by behavioral (CRS-R) and electrophysiological (MMN) assessments at four time points: before the treatment of HD-tDCS (T0), after a single session of HD-tDCS (T1), after the treatment of 7 days (T2) and 14 days (T3). In this study, any side effects of HD-tDCS were monitored and reported.

### ERP Assessment

#### MMN Paradigm

**FIGURE 1:**
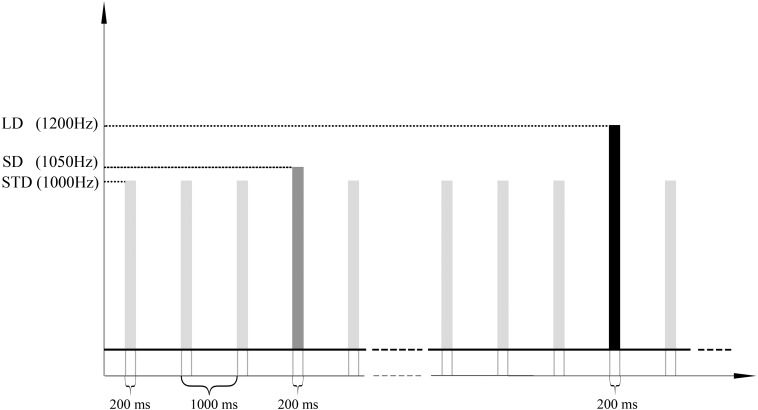
Stimulus sequences. 1000 pure sound stimuli (lasting for 200 ms) with SOA of 1000 ms were presented to a subject in order to elicit the MMN ERP response. The frequencies of standard, small deviant and large deviant stimuli were 1000 Hz, 1050 Hz, and 1200 Hz, and the numbers of trials were 800, 100 and 100. STD, SD, and LD represent the standard, the small deviant and the large deviant stimuli respectively.

We employed an oddball auditory paradigm which has been described previously (Wang et al., 2018) to elicit MMN. In this paradigm, a 1000 Hz pure sound was used as the standard stimulus, and two types of the deviant stimulus with different magnitude (a 1050 Hz and a 1200 Hz pure sound were employed as the small deviant stimulus and the large deviant stimulus respectively) were adopted. In the following, the standard stimulus will be called STD; deviants will be called SD (1050 Hz, the small deviant) and LD (1200 Hz, the large deviant). The paradigm consisted of 1000 sound stimuli lasting for 200 ms, and the stimulus onset asynchrony was 1000 ms. The stimuli were uninterrupted and pseudo-randomly presented with the probability of 0.8, 0.1, and 0.1 for STD, SD, and LD, and there were at least 3 STD between two consecutive deviants. Stimulus sequence was programed in the E-Prime 3.0 software (Psychology Software Tools, Pittsburgh, PA), and delivered through headphones. The experiments lasted approximately 17 min in total (Figure 1).

### ERP Data Acquisition

The ERP experiments were carried out at the patient’s bedside while patients were behaviorally awake. Scalp ERP was recorded at 28 electrodes(Fp1, Fp2, F3, Fc5, Fc1, Fc2, Fc6, F4, C3, C4, Cp1, Cp2, P3, P4, O1, O2, F7, F8, T3, T4, T5, T6, Fz, Cz, Pz, Poz, M1, and M2) according to 10/20 International System using a Nicolet amplifier by Natus Neurology Inc., The sampling rate was 1000 Hz, and the impedances of the electrodes were kept below 10 KΩ, and in most cases below 5 KΩ. Data were referenced online at CPz electrode and re-referenced offline with the mean potential at the mastoids on both sides.

### ERP Data Processing and Analysis

#### Preprocessing

EEG data were processed with the EEGLAB toolbox (Delorme and Makeig, 2004). The preprocessing was conducted on continuous EEG data. Raw data were visually inspected by an experienced data analyzer to remove significant artifacts caused by body movements, amplifier clipping, or bursts EEG activity. The nearby four good-quality channels interpolated channels with excessive artifacts. Basic filters embedded in EEGLAB were applied in the following order: 50Hz notch filter, 1Hz High-pass filter, and 30Hz low-pass filter (Widmann et al., 2015). Independent Component Analysis (ICA) was performed on filtered data using InfomaxICA algorithm (Lee et al., 1999) to spatially filter out the eye blink, horizontal eye movement, muscle activity, and electrocardiography artifacts. Regarding the scalp topography of the above independent components, for instance, both eye blink and movement have scalp projections centered on frontal electrodes, and eye blink represents in single-phase (negative or positive), while horizontal eye movement is anti-phase (one negative and one positive); the muscle activity centered on temporal electrodes (e.g., M1 and M2 electrodes); the electrocardiography artifacts closely approximate a diagonal linear gradient from left-posterior to right-anterior (Radüntz et al., 2017).

### Extracting Epochs, Averaging, and Calculating the Difference Waves

The preprocessed EEG data were segmented into epochs of 700ms, time-locked to stimulus onset, and included a pre-stimulus period of 100 ms (baseline). Then, the baseline was subtracted from each trial to ensure that all ERP segments had the same origin. Trials with amplitude exceeding 100 μV were excluded from averaging. To balance the signal-to-noise ratio, only the response to the standard tone immediately preceding the deviant tone was averaged. In consequence, four sweeps were obtained, SD, LD, the standard sweep proceeding the small deviant (SSD), and the standard sweep proceeding the large deviation (SLD). On average, there were 95, 94, 95, and 94 accepted epochs for SSD, SLD, SD, and LD, respectively.

Sabri and Campbell (2002) reported that slow-wave activities might attenuate the MMN component during wakefulness and proposed that a 3 Hz high-pass filter would permit the visualization of the MMN on the waking or sleeping MMN amplitude. Consequently, after averaging the responses for each measurement and subject, the ERPs were filtered between 3 and 30 Hz (Sabri and Campbell, 2002). Difference waveforms were computed by subtracting the averaged ERP elicited by the standard from that of the deviant.

### Criterions of Identifying and Quantifying MMN Properties

**FIGURE 2:**
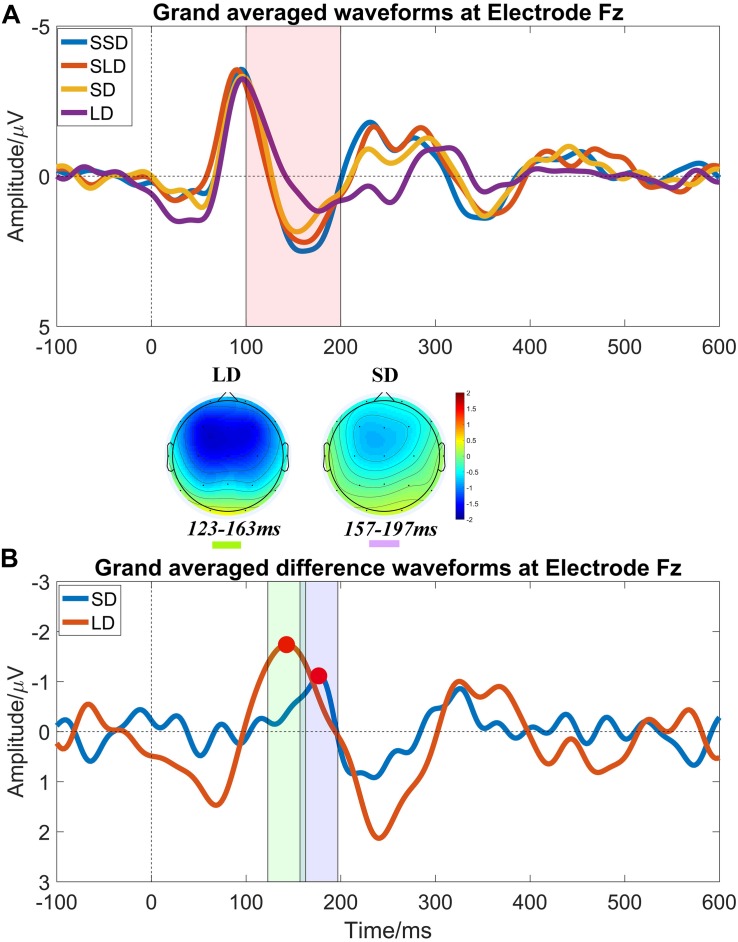
Auditory evoked ERP waveforms at electrode Fz in healthy controls at T0. **(A)** Grand averaged waveforms at electrode Fz, and it shows prominent N1 and P2 components. The pink shadow area indicates the latency interval between the peak of N1 component and the end of P2 component, and MMN properties should be measured within this area; **(B)** Grand averaged difference waveforms at electrode Fz and obvious MMN components peak in the pink shadow area. The mean value within the time window -20 ms to 20 ms centered on the latency of peak MMN component (marked by red points) was taken as MMN amplitude (the green shadow area and the purple shadow area indicate the measuring time window for LD and SD respectively). MMN topography of each stimulus shows a fronto-central focus, consistent with generators in the frontal lobe.

In this study, we collected the EEG data of 4 age-matched healthy volunteers as a control group using the same paradigm to set the criterions of identifying and quantifying MMN properties. Figure 2 illustrates the results of brain responses in healthy control group according to the above processing procedures.

The criterions of identifying MMN components were: (1) calculating the MMN properties (amplitudes and latencies) at electrode Fz (Figure 2A); (2) presence of N1 and P2 components is the pre-condition of measuring MMN properties; (3) searching the peaks of averaged difference waveforms (peaks of MMN component) in the latency interval between the peak of N1 component and the end of P2 component in the averaged waveforms (Figure 2B). Subsequently, the peak latencies in the averaged difference waveforms were regarded as the latencies of the MMN components; and the mean amplitude within a time window −20 ms to 20 ms centered on the latency of peak MMN component in the averaged difference waveform was taken as an MMN amplitude for further statistical analysis.

### Statistical Analysis

The statistical analysis was carried out on behavior data (CRS-R score) and ERP data (the amplitudes and latencies of MMN component) using IBM SPSS Statistics, Version 22. As for the behavior data, a one-way repeated measures analysis of variance (RMANOVA) was conducted to test the effects of Time (T0, T1, T2, and T3). Regarding the ERP data, two-way RMANOVAs were conducted to test the effects of Time (T0, T1, T2, and T3) and the magnitude of deviation (SD and LD) on MMN amplitude and latency. Greenhouse-Geisser Corrections were applied (the original degrees of freedom and corrected p-values are reported) if the Mauchly’s test of sphericity was not satisfied. Bonferroni corrections were carried out as *post hoc* analyses. Measures of effect sizes (Cohen’s d and partial eta-squared) are interpreted using Cohen (2013) guidelines. Correlation analysis was performed between CRS-R scores and MMN amplitudes across subject (4 sessions × 11 subjects) by repeated measures correlation using R (Bakdash and Marusich, 2017). The significance level was set at 0.05.

## Results

### Effect of the HD-tDCS Treatment as Measured by CRS-R

**FIGURE 3:**
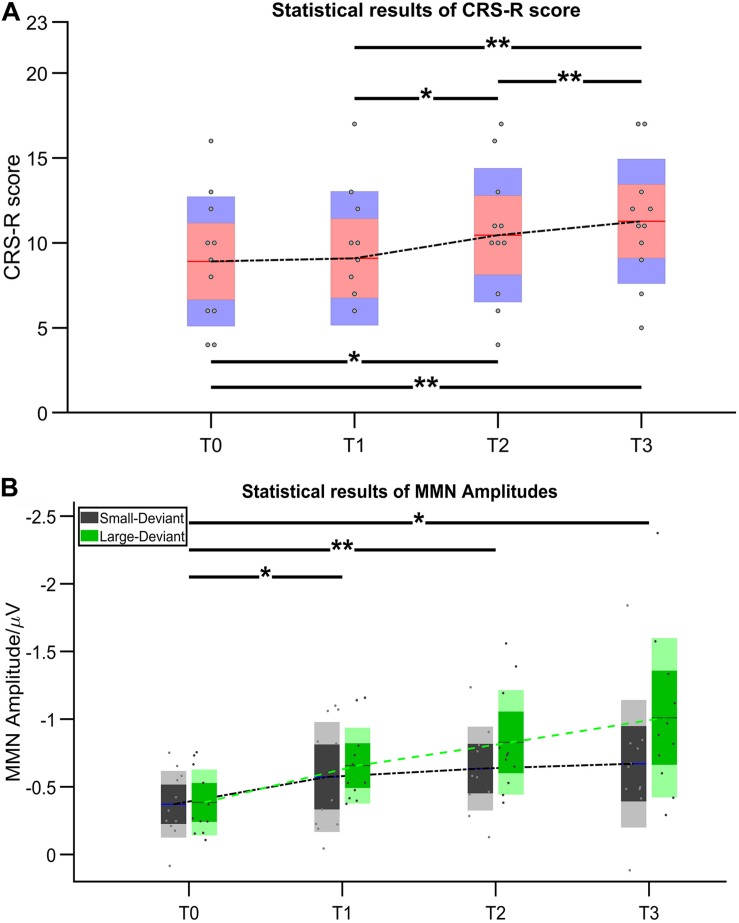
Statistical results of **(A)** CRS-R score and **(B)** MMN amplitude. Asterisk indicates significant differences (*p ≤ 0.05, **p ≤ 0.01).

After 14-day HD-tDCS stimulations, 11/11 patients showed an increase in the CRS-R total scores (Table 2). In addition, the Time (T0, T1, T2, T3) RMANOVA showed a statistically significant main effect of Time [F (1.19, 11.88) = 18.97, *p* = 0.001, η_p_^2^ = 0.655]. *Post hoc* revealed that, compared with the baseline (T0 with mean = 8.909), statistically significant improvements were observed after 7-day (T2 with mean = 10.455, *p* = 0.016, Cohen’s d = 1.324) and 14-day treatment (T3 with mean = 11.237, *p* = 0.004, Cohen’s d = 2.067), whereas there was no statistically significant improvement after single session of stimulation (T1 with mean = 9.091, *p* > 0.05, Cohen’s d = 0.156). In addition, significant differences in the pairs among T1, T2, and T3 (all *p*’s < 0.05) were found. The mean difference of CRS-R score between T2 and T1 was 1.364 (*p* = 0.023, Cohen’s d = 1.150); the mean difference of CRS-R score between T3 and T1 was 2.182 (p = 0.007, Cohen’s d = 1.904); and the mean difference of CRS-R score between T3 and T2 was 0.818 (*p* = 0.007, Cohen’s d = 0.714), detail in Figure 3A.

**TABLE 2:** CRS-R scores and MMN amplitudes among four-time measurements.

**Patient**	**T0 CRS-R score SD/LD**	**T1**	**T2**	**T3**	**Changes in diagnosis**
1	6 −0.21/−0.25	6 −0.83/−0.67	6 −0.81/−0.79	7 −0.50/−0.82	Remained MCS−
2	10 −0.25/−0.15	10 −0.33/−0.48	11 −0.13/−0.38	12 0.12/−0.42	Remained MCS−
3	8 −0.18/−0.16	8 −0.04/−0.42	10 −0.46/−0.75	11 −0.41/−0.60	Remained MCS−
4	9 −0.58/−0.53	9 −1.06/−0.53	10 −0.57/−1.39	10 −0.48/−1.33	Remained MCS−
5	6 −0.32/−0.67	7 −1.07/−1.14	10 −0.80/−1.19	11 −0.78/−0.88	Remained MCS−
6	4 −0.55/−0.24	4 −0.23/−0.73	7 −0.91/−0.70	9 −0.85/−1.12	VS elevated to MCS−
7	4 −0.42/−0.73	4 −0.40/−0.53	4 −0.28/−0.65	5 −0.48/−0.97	Remained VS
8	13 −0.65/−0.76	13 −0.82/−0.80	16 −1.24/−1.56	17 −1.84/−2.37	MCS− elevated to MCS +
9	12 −0.75/−0.37	12 −1.10/−1.16	13 −0.76/−0.73	13 −0.82/−1.57	MCS− elevated to MCS +
10	10 0.08/−0.11	10 −0.19/−0.40	11 −0.58/−0.44	12 −0.67/−0.29	Remained MCS−
11	16 −0.25/−0.27	17 −0.22/−0.37	17 −0.44/−0.53	17 −0.65/−0.73	MCS- elevated to MCS +

### Effect of the HD-tDCS Treatment as Measured by MMN

**FIGURE 4:**
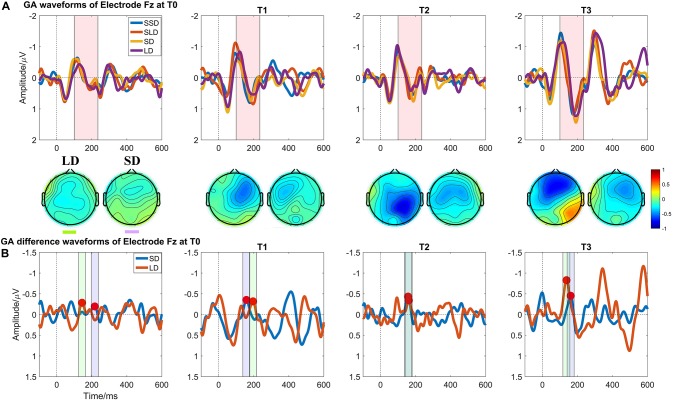
Auditory evoked ERP waveforms at Electrode Fz in patients with DOCs at four measuring time points. **(A)** Grand averaged waveforms at Electrode Fz; the pink shadow area indicates the latency interval between the peak of N1 component and the end of P2 component; **(B)** Grand averaged difference waveforms at Electrode Fz and the green shadow area, and the purple shadow area indicate the measuring time window for LD and SD respectively.

Figure 4 illustrates the ERP results in patients group at four time points. Regarding the MMN amplitudes, the Time (T0, T1, T2, T3) ^∗^ deviation magnitude (SD, LD) RMANOVA revealed a significantly main effect of Time [F (3, 30) = 8.850, *p* < 0.001, η_p_^2^ = 0.470] and a significantly main effect of deviation magnitude [F (1, 10) = 22.437, *p* = 0.001, η_p_^2^ = 0.692], whereas no significantly interaction effect between Time and Stimuli was observed [F (1.975, 19.746) = 2.360, *p* = 0.121, η_p_^2^ = 0.191]. As for the significantly main effect of Time, *post hoc* analysis with Bonferroni corrections showed that compared with the baseline (T0 with mean = −0.378 μV), statistically significant improvements were observed after a single session of stimulation (T1 with mean = −0.615 μV, *p* = 0.048, Cohen’s d = 2.857), 7-day (T2 with mean = −0.731 μV, *p* = 0.004, Cohen’s d = 4.285) and 14-day treatment (T3 with mean = −0.840 μV, *p* = 0.011, Cohen’s d = 3.943). Although no significant differences in the pairs among T1, T2, and T3 (all *p*’s > 0.05) were found, the mean values on T1, T2, and T3 were greater across time. The mean difference of MMN amplitude between T2 and T1 was −0.117 (*p* = 0.082, Cohen’s d = 1.202); the mean difference of MMN amplitude between T3 and T1 was −0.226 (*p* = 0.130, Cohen’s d = 1.765); and the mean difference of MMN amplitude between T3 and T2 was −0.109 (*p* = 0.085, Cohen’s d = 0.857), detail in Figure 3B. In addition, SD (mean = −0.562 μV) was smaller than LD (mean = −0.720 μV) with Bonferroni corrected *post hoc* comparison (*p* = 0.001, Cohen’s d = 1.725). The detail in Table 2.

Regarding the MMN latencies, the Time (T0, T1, T2, T3) ^∗^ deviation magnitude (SD, LD) RMANOVA revealed a statistically significant main effect of deviation magnitude [F (1, 10) = 7.897, *p* = 0.018, η_p_^2^ = 0.441], whereas no statistically significant interaction effect between Time and Stimuli [F (3, 30) = 0.728, *p* = 0.543, η_p_^2^ = 0.068] or main effect of Time [F (3, 30) = 1.118, *p* = 0.357, η_p_^2^ = 0.101] was observed.

### Correlation Analysis

**FIGURE 5:**
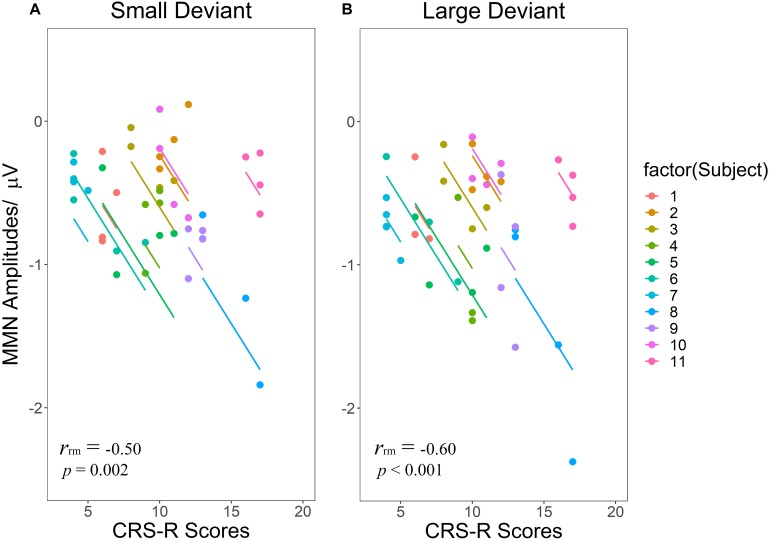
Scatterplots of the linear correlations between CRS-R scores and MMN amplitudes elicited by SD **(A)** and LD **(B)**. Repeated measurements for each patient are plotted in the same color pattern. Linear regression lines are correspondent to repeated measurements within patients. SD and LD represent the small deviant and the large deviant stimuli respectively.

Regarding the correlations between CRS-R total scores and MMN amplitudes (Figures 5A,B), both LD [*r_rm_* = −0.60 (95% CI: −0.78– −0.31) (*p* < 0.001)] and SD [*r_rm_* = −0.50 (95% CI: −0.72– −0.18) (*p* = 0.002)] showed significant negative correlations, namely as the CRS-R scores increases, the absolute value of MMN becomes larger.

## Discussion

The overarching goal of this study was to evaluate the clinical efficacy of long-term HD-tDCS on improving consciousness in patients with DOCs and determine the potential of MMN to access the LOC in patients with DOCs. In this study, the clinical behavior outcome was assessed by the CRS-R scale, and we adopted an oddball auditory paradigm with two frequency-deviant stimuli to elicit MMN. To our best knowledge, this is the first study combining the CRS-R scale, and MMN evaluates the clinical efficacy of long-term HD-tDCS in patients with DOCs. The main findings can be summarized as follows: (i) long-term HD-tDCS has a statistically significant effect on recovering of the level of consciousness measured by CRS-R scale in patients with MCS (9/11) and VS (2/11) after 7-day and 14-day treatment; (ii) as the treatment conducting, MMN amplitudes elicited by both large and small deviant stimuli show significantly continuous increases compared with baseline (T0); (iii) significant negative correlations were found between CRS-R scores and MMN amplitudes elicited by both large and small deviant stimuli.

The enhancement of total CRS-R scores with anodal HD-tDCS over precuneus in this study replicates the significant results found in our previous pilot study examining the effects of HD-tDCS (Guo et al., 2019). Interestingly, all enrolled patients (9 MCS and 2 VS) showed an increase in CRS-R total scores after 14 days of stimulation, whereas previous studies demonstrated that tDCS might transiently improve signs of consciousness only in MCS (Thibaut et al., 2014). In this study, we speculated that three experimental issues might contribute to the current treatment effect. Firstly, regarding the site of tDCS stimulation, differentiate from the majority of studies showing the clinical efficacy of conventional tDCS on left DLPFC, we conducted stimulations on precuneus, which is thought to play essential functions in conscious processes (Laureys and Schiff, 2012). Maquet et al. (1999) reported that deactivation of the precuneus is considered to be a critical metabolic feature of altering the state of consciousness. And the precuneus is among the first regions of the brain to resume its activity if patients regain consciousness (Cavanna and Trimble, 2006). Additionally, a recent study reported that repetitive TMS over left angular gyrus connected with the precuneus improved the CRS-R total score in MCS patients (Legostaeva et al., 2019). As a consequence, it could be speculated that tDCS over precuneus effectively altered the metabolism, which leads to improvements in consciousness as assessed by changes in CRS-R total scores.

Secondly, such results might benefit from the enhanced characteristics of HD-tDCS, including more focal stimulation and higher spatial accuracy. Compared with conventional tDCS, HD-tDCS may offer the opportunity for the induction of neuroplasticity in the primary motor cortex in healthy controls (Nitsche et al., 2007; Kuo et al., 2013) and lead to slightly higher naming accuracy in patients with aphasia (Richardson et al., 2015). Notable, neuroplasticity induced by HD-tDCS would extend the duration of reaching the peak of excitability alteration and after-effects (Nitsche et al., 2007; Kuo et al., 2013), which allows tolerant time for patients to be assessed in a high stimulated efficacy. Thirdly, significantly behavior improvements could not be observed until seven days or later and significant increases of CRS-R score were found from T1 to T2 and T2 to T3, which might indicate that cumulative effects of repeated sessions and long-term HD-tDCS might contribute to the recovering of consciousness. This explanation is supported by studies that showed multiple repeated sessions of tDCS to improve the clinical status of patients, including DOCs (Angelakis et al., 2014), stroke (Boggio et al., 2007), and major depression (Loo et al., 2012). Moreover, the parameters of HD-tDCS protocol could be further optimized in the following studies, including current intensity, number of repeated sessions, and number of stimulating days. Taking advantage of the above clinical factors, our study demonstrates that long-term HD-tDCS on precuneus could improve the behavior outcome as measured by the change of the CRS-R scale.

Impairment in auditory discrimination has been repeatedly reported in patients with DOCs. MMN, as an electrophysiological index, reflects the auditory discrimination, which is considered to be a predictor of awakening (Kane et al., 1993; Fischer et al., 1999; Wijnen et al., 2007; Daltrozzo et al., 2009). At present, the MMN amplitudes increased across time when compared to baseline. Although no significant differences in the pairs among T1, T2, and T3 were found, the mean values on T1, T2, and T3 were greater across time. According to Cohen’s guidelines, effect sizes can be considered small (d = 0.2 and eta-squared = 0.01), medium (d = 0.5 and eta-squared = 0.06), or large (d = 0.8 and eta-squared = 0.14), and all Cohen’s ds of MMN amplitudes comparisons between T1 and T2, T2 and T3, T1 and T3, were bigger than 0.8, which indicated a large effect of the treatment Time on MMN amplitudes. Interestingly, MMN has been attributed to neural generators within the temporal, and frontal lobes (Fishman, 2014); nevertheless, our results suggest that performing tDCS on precuneus could lead to a continuous increase of MMN amplitudes compared with baseline. Precuneus and medial prefrontal cortex are linked to the default mode network (DMN), where connectivity decreased in patients with DOCs (Norton et al., 2012; Koenig et al., 2014); primarily, fronto-parietal circuits result to be connectively disrupted in patients with DOCs (Cavinato et al., 2015). Koch et al. reported that TMS targeting the precuneus leads to a modification of functional connections between the precuneus and medial frontal areas within the DMN in patients with Alzheimer’s disease (Koch et al., 2018). Furthermore, previous studies demonstrated that long-term anodal tDCS lasting effects may be mediated by synaptic pathways through N-methyl-D-aspartate (NMDA) receptor activity (Liebetanz et al., 2002; Nitsche et al., 2003), which plays an important role in modulating MMN-indexed auditory discrimination (Impey et al., 2017). Considering connectivity and synaptic plasticity together, Naro et al. (2015) reported that anodal tDCS over orbitofrontal cortex increased primary motor cortex excitability and modulated premotor-motor connectivity in some DOC patients, Hence, we speculated that anodal tDCS over precuneus modulates the excitatory and inhibitory neurotransmitter activity and enhance the connectivity within the DMN contributing to the increase of MMN amplitudes, which indicated consciousness recovery.

Patients with more ameliorated positive symptoms showed an increased MMN amplitudes in the present study, which has been reported previously (Wijnen et al., 2007; Wang et al., 2018). Different from other MMN paradigms in patients with DOCs, the present study employed a multi-deviant MMN paradigm in four-time measures. Present results indicate that the LD (200Hz difference) seems to be beyond the discrimination threshold in most patients with DOCs over the therapy process. As for the comparison between LD and SD, the latter shows a weak correlation result, which might suggest that these patients couldn’t regain comparable auditory discrimination to discriminate the SD (50Hz difference). The result might suggest that auditory discrimination threshold varies in different levels of consciousness. Furthermore, we are optimizing the MMN paradigm to improve the clinical efficacy in patients with DOCs, mainly including the stability of deviant type (frequency, duration, intensity, location, gap, etc.), stimulus deviance and the time of the experiment.

The present study still has several limitations. Firstly, no control group was enrolled in this study, which could not exclude other factors that might contribute to treatment effects. The treatment paradigm could be further optimized. Secondly, the statistical sample size was small, and even not balanced between diagnosis (2 VS and 9 MCS) and etiology (8 Hemorrhage, 2 TBI, 1 anoxia and 1 stroke), which reduced the power of the study. With additional data, we could further investigate the effects of etiology, post-injury duration, and so forth. Moreover, our hypothesis about the SD, which might be potentially used in patients with high LOC, possibly MCS + patients with a lower discrimination threshold, or a longer-term stimulation, could be further tested. Furthermore, this study lacked a randomized cross-over design and follow-up assessment to verify the long-term effect of HD-tDCS. Currently, we only employed EEG as a functional neuroimaging tool. However, some of the results may benefit from the functional connectivity and/or metabolism within the brain regions, and we can further utilize multi-modality technology, including the fMRI and PET to explore the mechanisms.

## Data Availability Statement

The datasets generated for this study are available on request to the corresponding author.

## Ethics Statement

The studies involving human participants were reviewed and approved by The ethics committee of the Zhengzhou Central Hospital. The patients/participants provided their written informed consent to participate in this study. Written informed consent was obtained from the individual(s) for the publication of any potentially identifiable images or data included in this article.

## Author Contributions

FC, YG, JH, and HZ designed the study. JL, ZG, YL, and TZ collected the data. XW and YZ analyzed the data. XW and YG wrote and edited the manuscript.

## Conflict of Interest

The authors declare that the research was conducted in the absence of any commercial or financial relationships that could be construed as a potential conflict of interest.
